# A pilot study of ex-vivo MRI-PDFF of donor livers for assessment of steatosis and predicting early graft dysfunction

**DOI:** 10.1371/journal.pone.0232006

**Published:** 2020-05-14

**Authors:** Sanjaya K. Satapathy, Humberto C. Gonzalez, Jason Vanatta, Andrew Dyer, Wesley Angel, Simonne S. Nouer, Mehmet Kocak, Satish K. Kedia, Yu Jiang, Ian Clark, Nour Yadak, Nosratollah Nezakagtoo, Ryan Helmick, Peter Horton, Luis Campos, Uchenna Agbim, Benedict Maliakkal, Daniel Maluf, Satheesh Nair, Hollis H. Halford, James D. Eason

**Affiliations:** 1 Division of Transplant Surgery, Methodist University Hospital Transplant Institute, Memphis, TN, United States of America; 2 Department of Surgery, University of Tennessee Health Science Center, Memphis, TN, United States of America; 3 Division of Hepatology, Sandra Atlas Bass Center for Liver Diseases, Northwell Health/ North Shore University Hospital, Manhasset, New York, United States of America; 4 Wayne State University School of Medicine/Henry Ford Health System, Detroit, MI, United States of America; 5 Center for Abdominal Transplantation, Cleveland Clinic Florida, Weston, FL, United States of America; 6 Department of Radiology, Methodist University Hospital, Memphis, TN, United States of America; 7 Department of Preventive Medicine, University of Tennessee Health Sciences Center, Knoxville, Tennessee, United States of America; 8 School of Public Health, University of Memphis, Memphis, TN, United States of America; 9 Department of Pathology, University of Tennessee Health Science Center, Memphis, TN, United States of America; Linköping University, SWEDEN

## Abstract

**Background:**

The utility of ex vivo Magnetic resonance imaging proton density fat fraction (MRI-PDFF) in donor liver fat quantification is unknown.

**Purpose:**

To evaluate the diagnostic accuracy and utility in predicting early allograft dysfunction (EAD) of ex vivo MRI-PDFF measurement of fat in deceased donor livers using histology as the gold standard.

**Methods:**

We performed Ex vivo, 1.5 Tesla MRI-PDFF on 33 human deceased donor livers before implantation, enroute to the operating room. After the exclusion of 4 images (technical errors), 29 MRI images were evaluable. Histology was evaluable in 27 of 29 patients. EAD was defined as a peak value of aminotransferase >2000 IU/mL during the first week or an INR of ≥1.6 or bilirubin ≥10 mg/dL at day 7.

**Results:**

MRI-PDFF values showed a strong positive correlation (Pearson’s correlation coefficient) when histology (macro-steatosis) was included (*r =* 0.78, 95% confidence interval 0.57‐0.89, p<0.0001). The correlation appeared much stronger when macro plus micro-steatosis were included (*r =* 0.87, 95% confidence interval 0.72‐0.94, p<0.0001). EAD was noted in 7(25%) subjects. AUC (Area Under the Curve) for macro steatosis (histology) predicted EAD in 73% (95% CI: 48–99), micro plus macro steatosis in 76% (95% CI: 49–100). AUC for PDFF values predicted EAD in 67(35–98). Comparison of the ROC curves in a multivariate model revealed, adding MRI PDFF values to macro steatosis increased the ability of the model in predicting EAD (AUC: 79%, 95% CI: 59–99), and addition of macro plus micro steatosis based on histology predicted EAD even better (AUC: 90%: 79–100, P = 0.054).

**Conclusion:**

In this pilot study, MRI-PDFF imaging showed potential utility in quantifying hepatic steatosis ex-vivo donor liver evaluation and the ability to predict EAD related to severe allograft steatosis in the recipient.

## Introduction

Liver Transplantation of steatotic livers has been associated with an increased risk of graft failure or impaired graft function as many of these organs suffer from ischemia-reperfusion injury (IRI) after transplantation[1]. There is an ongoing debate regarding what constitutes significant and acceptable risk in terms of the amount and type of fat in a steatotic graft. Typically, grafts with 60% steatosis are not transplanted, while those with 30–60% steatosis when transplanted have been associated with poor results, such as impaired graft function, and decreased graft and patient survival. It has been recommended though that organs with 30% steatosis only are used if other factors are controlled (i.e., donor age <40 years, short cold ischemia time of< 5 hr, and non-circulatory cause of death)[2]. Given the growing demand for LT and the need to use increasingly marginal donor organs, information is needed on how best to assess the fat contents in the liver. Liver biopsy currently remains the only method to quantify hepatic steatosis. Unfortunately, liver biopsy only samples 1/100,000^th^ of the liver and is fraught with significant sampling variability. Inter-observer variation also remains a concern. As such, there is a need for innovative ways to better quantify hepatic fat to improve the quality of the donor, increase the donor pool, and potentially impact the outcome after LT.

Proton density fat fraction (PDFF) measurement is an MRI-based method for quantitatively assessing hepatic steatosis and is available from several manufacturers of MRI scanners as an option. Unlike older fat suppression and chemical shift techniques, PDFF accounts for several technical and biological confounders that affect the apparent fat fraction. PDFF produces similar results to multi-voxel MR spectroscopy and can be acquired much faster[3]. The fat fraction map generated by the software is quantitative and reflects the proton density of triglyceride fat in the liver. This is calculated by dividing the triglyceride proton density by the sum of the water and triglyceride proton densities on a voxel-by-voxel basis. MRI-determined PDFF correlates with histologically determined steatosis grade in patients with NAFLD (Nonalcoholic fatty liver disease)[4]. However, the utility of *ex vivo MRI determined fat quantification* for donor selection has not been studied.

We sought to evaluate if donor liver fat fraction measurement using ex vivo Magnetic Resonance Imaging (ex vivo MRI PDFF) is a useful tool in liver allograft selection. We hypothesized that donor liver fat fraction measurement using Magnetic Resonance Imaging (MRI PDFF) correlates with histology-determined hepatic steatosis in the deceased liver. We also sought to evaluate the utility of ex vivo MRI-determined Proton density fat fraction (PDFF) measurement in predicting post-transplant outcome compared to histology-determined hepatic steatosis on donor liver biopsy.

## Methods

After the procurement of donor liver, liver biopsies were performed at the donor hospital, and the donor histopathology was interpreted by the pathologist for suitability for a liver transplant. In the event biopsy data was not available from the donor hospital, we used biopsy results of the transplant center pathologist. These results were reviewed by the procuring surgeons, and if considered suitable, accepted for transplantation. The results of the histopathology are available through the United Network for Organ Sharing donor portal. All livers considered suitable for transplant were then subjected to an ex-vivo MRI scan, while still enclosed in the transport box and MR-PDFF assessment performed.

### MRI protocol

**Fig 1:**
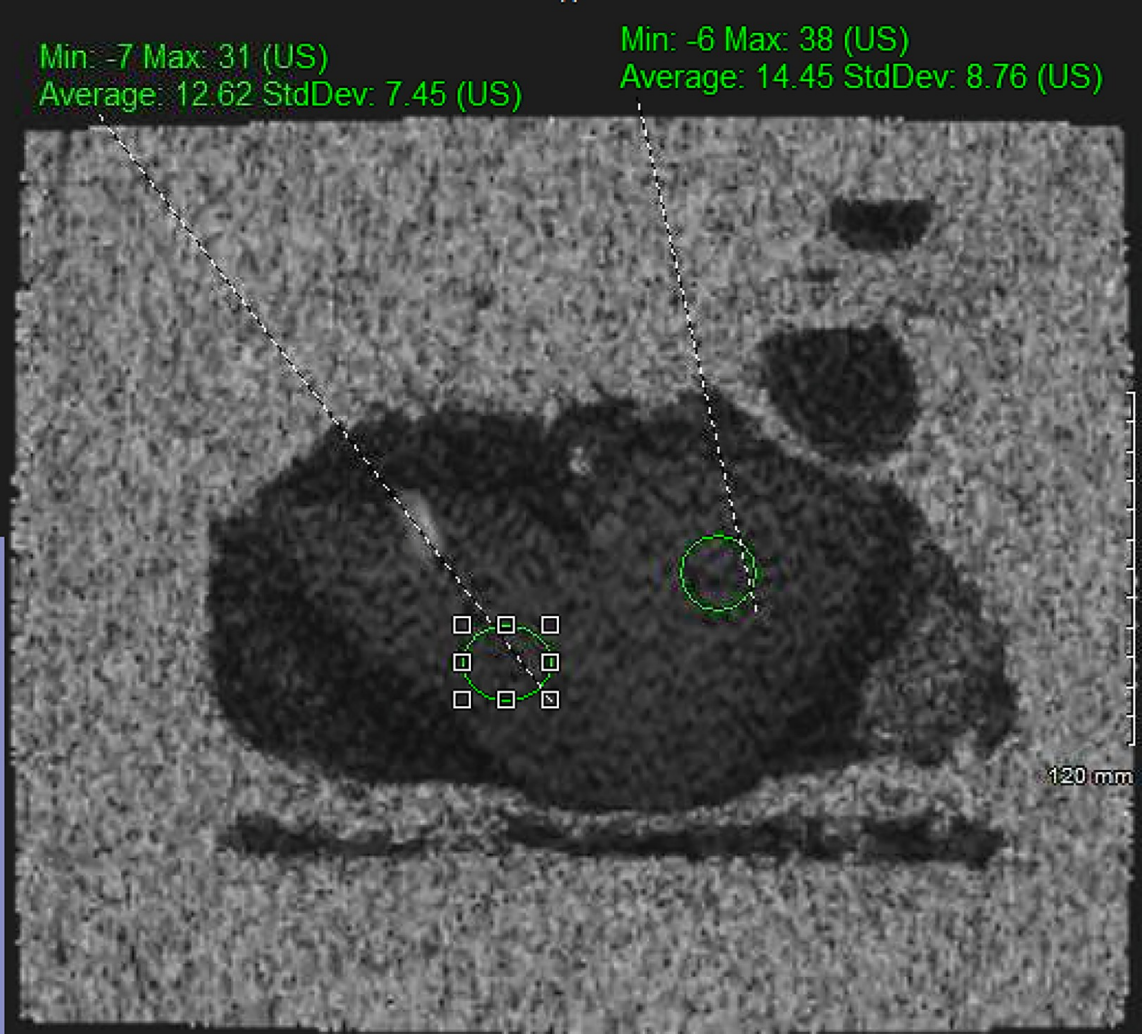
Representative T2 weighted images with the fat fraction map in region of interest (ROI) to delineate different segments.

To measure hepatic PDFF, a state-of-the-art MR imaging technique was performed. The protocol utilized an MR-sequence product for the creation of PDFF maps for each liver (IDEAL-IQ; GE Healthcare). Imaging was performed at 1.5T on GE Optima MR450w. Axial T2 single-shot fast spin-echo sequences (SSFSE) were utilized for anatomic correlation with PDFF maps. The technique is composed of a 3D fast spoiled gradient-echo sequence with a low flip angle (FA) to minimize T1 bias, and it acquires multiple echoes to calculate triglyceride fat and water in each pixel based on their phase differences. Data obtained at each of the echo times are then passed to a nonlinear least-squares fitting algorithm that estimates and corrects T2* effects. This allows for more accurate modeling of the fat signal as multiple spectral peaks and estimates fat and water proton densities from which the fat content is calculated [5]. Using analysis software, a mathematical model was then applied pixel by pixel on the source images to generate parametric PDFF maps that depicted the quantity and distribution of fat throughout the entire liver. Imaging PDFF was measured in regions of interest (ROI) approximately 300–400 mm^2^ in area placed on the PDFF parametric maps, avoiding vessels, bile ducts, lesions and artefacts. ROIs were placed in all 9 of the liver segments on the MR exams (counting segment 4A and 4B as separate segments). Two experienced radiologists (AD-15+ years’ and WA- 5+ years’ experience) who were blinded of the clinical and histological information interpreted the PDFF values independently, and the per-liver PDFF measurements were then averaged. A representative measurement on two different segments of the liver by ex-vivo MRI is presented in Fig 1.

This approach required a dedicated well-trained MRI staff, and a research coordinator who was available 24 hrs and 7 days (on phone) to coordinate the availability of the MRI room, and staffing of MRI technician for the MRI imaging. In general, as soon as an organ was available, the transplant coordinator notified the principal investigator (SKS). The PI and the research coordinator then coordinated the timing of the MRI based on an approximate organ arrival time with the MRI technician. The center has two dedicated state of the art MRI rooms, and one the room was kept open in anticipation of the organ arrival at least 30 minutes prior to organ arrival to avoid any delay in transportation-related time to the MRI imaging enroute to the operating room.

The decision to proceed with liver transplant was not based on the interpretation of the fat quantification as determined by MRI-PDFF methods, and the transplant surgeons were blinded to these results at the time of the liver transplant.

Outcome assessment:

To objectively evaluate the differences in the post-transplant outcome, we specifically evaluated the following outcomes:

1. EAD (Early allograft dysfunction).
2. Bilirubin level, Alanine Aminotransferase (ALT), aspartate aminotransferase (AST), Alkaline phosphatase (ALP), and INR in the immediate post-transplant period.
3. Need for operations post-transplant within 90 days.
4. The length of ICU stays in the immediate post-transplant period.
5. Length of hospitalization.

We used a previously validated definition of early allograft dysfunction (EAD) described by Olthoff in the present study. EAD was thus defined as the presence of 1 or more of the following variables: [1] bilirubin ≥ 10 mg/dL on post-operative day 7, [2] INR ≥ 1.6 on postoperative day 7, and [3] an aminotransferase level (ALT or AST) ≥ 2000 IU/mL within the first 7 postoperative days[6].

The Institutional Review Boards of the University of Tennessee Health Science Center approved the study and informed consent was obtained from all participants (organ recipients) before inclusion in the study. Institutional Review Boards of the University of Tennessee Health Science did not request additional consent from the donor as the organ after procurement was de-identified, and any clinical information related to the donor is linked with a code.

### Statistical analysis

Baseline recipient characteristics were summarized at the time of LT and presented as percentages (rounded to the nearest whole number wherever applicable) for categorical variables and mean ± standard deviation (SD) for continuous variables.

The accuracy of MRI‐PDFF was assessed through linear regression with MRI‐PDFF, and histologic steatosis quantification. Measures of association of the histology determined hepatic steatosis, and the MRI PDFF determined hepatic fat was performed using the Pearson product-moment correlation test. Pearson's correlation coefficients (*r*) with 95% confidence intervals (CI) and significance levels (*P* values) were computed to express the degree of linear association between measures. We defined correlation coefficients as “weak” if r = 0.10 to 0.39, “moderate” if r = 0.4–0.69, “strong” if *r*  = 0.70 to 0.89 and as “very strong” if *r >* 0.9[7].

We estimated interclass coefficient of reliability [ICC (95% CI)], and coefficient of within-subject variance (95% CI) for the MRI readouts from the two independent radiologists who are blinded of the clinical information, and pathology readouts for the two independent local pathologists as well as the readouts from the procurement site pathologists using the SAS ICC9 Macro. [8] We also calculated the area under ROC curves in predicting EAD based on degree of hepatic steatosis and compared the results of hepatic steatosis quantification based on histology of the donor liver, and MRI-PDFF measurement.

Statistical graphics and computations were performed using SAS Version 9.4 (Cary, NC, USA). Differences were considered statistically significant if the 95% CIs did not overlap or if *P* < 0.05.

## Results

After the exclusion of 4 images due to technical inaccuracies, 29 MRI images were evaluable. Histology was available in 28/29 patients. These 28 patients formed the analysis cohort. Only 23 of the 28 biopsy slides included in the study were accessible for a re-read by local pathologists. All the 5 inaccessible slides had < 5% steatosis (reported as 0% in 4, and 5% in one). The internal pathologist on the second review described, 6(26%) as poor quality, 7(30%) as adequate, and the rest 10(43%) as a good quality wedge or core liver biopsies. An average of 2.6 slides was examined (range: 1–6).

The mean age of the OLT recipients included in the study was 59±11years, with 14(50%) males, and the predominant majority were of Caucasian ethnicity. The mean BMI of the recipient was 27.6±6 Kg/m^2^, 6(21%) were diabetic, and 14(50) were hypertensive. Pre-transplant liver function tests, INR, serum Cr, donor characteristics, immunosuppression regimen in the immediate post-LT period is presented in Table 1. The mean MRI-PDFF of our cohort, expressed as %, was 6.4± 2.7 with a median of 5.4% (interquartile range: 4.4%,8.1%). The mean MRI scan time, expressed in minutes, was 2.8±1.5, with a median of 2 minutes (interquartile range:2,3). MRI to organ-in-room time, expressed in minutes, was 12.4±4, with a median time of 11 minutes (interquartile range: 9,16). A summary of the histology findings and PDFF values is presented in S1 Table.

**Table 1:** Clinical and demographic characteristics of the donor and recipients.

Recipient characteristics	All patients (n = 28)
Age at Transplant	59±11
Gender (Male)	14(50)
Race (Caucasian)	21(75)
BMI	27.6±6
Diabetes	6(21)
Hypertension	14(50)
Bilirubin level	5.7±7.8
AST (IU/mL)	58±47
ALT (IU/mL)	38±22
ALP (IU/mL)	158±94
INR	2±0.8
Creatinine (mg/dL)	1.5±1
**Donor characteristics**	
Donor Age	35±16
Donor BMI	28±9
Donor Gender (Male)	20(71)
Donor Type (DCD)	10(36)
Donor CIT	282±88
Immunosuppression (beyond 1 week)	
Tacrolimus	28(100)
Mycophenolic acid	23(82)
m-TOR inhibitor	5(18)
**MRI data**	
MRI PDFF Median (IQR)	6.4± 2.7 5.4(4.4,8.1
MRI scan time (minutes) Median (IQR)	2.8±1.5 2(2,3)
MRI to organ-in-room time (minutes)	12.4±4 11 (9,16)

### Accuracy of MR PDFF in predicting allograft steatosis by histology

**Fig 2:**
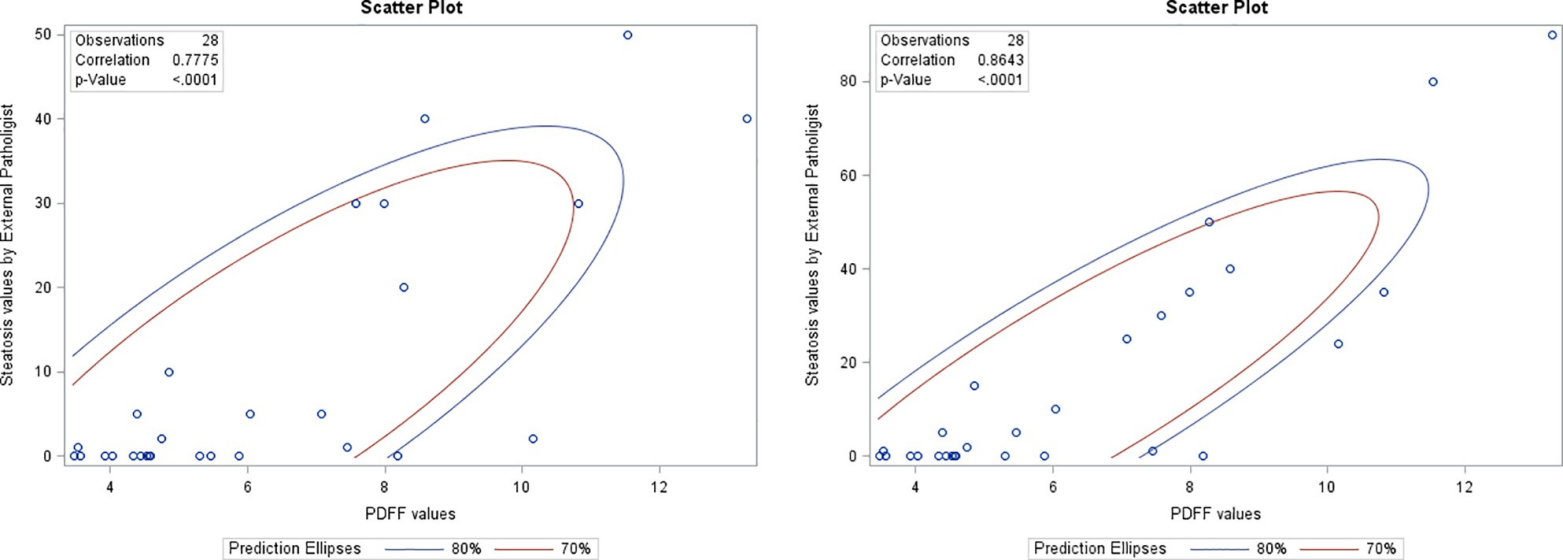
A. Correlation of MRI-PDFF with histology (external pathologist read out) when only macro steatosis was included. B. Correlation of MRI-PDFF with histology (external pathologist-read out) when macro plus micro steatosis was included.

A Pearson product-moment correlation coefficient was computed to assess the relationship between MRI-PDFF values and hepatic steatosis % on histology as measured by the procurement site pathologist using macro steatosis read outs. MRI-PDFF values showed a strong positive correlation (Pearson’s correlation coefficient) with histology when only macro steatosis was included (*r =* 0.78, 95% confidence interval 0.57‐0.89, p<0.0001), and the results are summarized in a scatterplot (Fig 2A). The correlation appeared much stronger when both macro plus micro-steatosis were included (*r =* 0.87, 95% confidence interval 0.72‐0.94, p<0.0001, Fig 2B). Overall, there was a strong, positive correlation of hepatic steatosis measured by MRI PDFF and liver histology. Increases in MRI PDFF correlated with increases in hepatic steatosis as determined by histology. Similar correlation of the mean MRI PDFF value was noted for macro-steatosis read-out by pathologist A (r = 0.66, 95% confidence interval 0.33‐0.84, p = 0.0007, S1 Fig), and pathologist B (r = 0.73, 95% confidence interval 0.46‐0.88, p<0.0001, S2 Fig), as well as for macro+micro-steatosis read-out for pathologist A (r = 0.78, 95% confidence interval 0.55‐0.90, p<0.0001, S3 Fig), and pathologist B (r = 0.78, 95% confidence interval 0.54‐0.90, p<0.0001, S4 Fig).

Further analysis using a linear regression model demonstrated a significant regression equation when Histology (using macro steatosis read-out by the procurement site pathologist) was used as the outcome variable and MRI PDFF as the predictive variable: (F (1, 26) = 39.73, P<0.0001), with an R^2^ of 0.61, S5 Fig. Every unit increase in MRI PDFF values resulted in 4.49 ± 0.7% increase in %macro steatosis. This association was even much stronger when both macro and micro-steatosis were included: (F (1, 26) = 76.74, P<0.0001, S6 Fig), with an R^2^ of 0.75. Every unit increase in %MRI PDFF values resulted in 8± 0.9% increase in %macro+micro steatosis (p< 0.0001). A similar significant regression equation was obtained when macro-steatosis read-out by the local pathologist A and B was used as the outcome variable and mean MRI PDFF as the predictive variable (S5 & S6 Figs).

**Fig 3:**
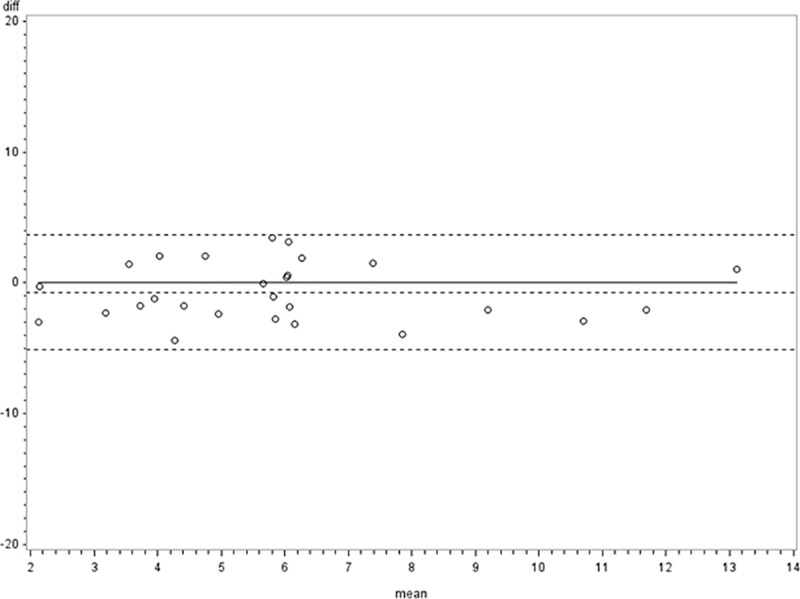
Mean differences in the PDFF values for two radiologists plotted across 95% CI.

The agreement between Internal Pathologists as well as the agreement between Internal & External pathologists on macro-steatosis, micro-steatosis, and combined macro & microsteatosis is shown in Table 2. We noted an excellent reliability between the internal pathologist Intraclass Correlation Coefficient (ICC) (95% CI); 0.89 (0.77, 0.95)], but the agreement was somewhat less reliable between the internal and external pathologists for macro-steatosis readout [ICC (95% CI); 0.46 (0.25, 0.69)]. We have also shown the estimated coefficient of reliability [ICC (95% CI)], and estimated coefficient of within-subject variance (95% CI) for the read-outs of PDFF values by two independent experienced radiologists (AD with 10+ years of experience, and WA with 5+ years of experiences) who were blinded of the clinical information (Table 3). We have shown excellent reliability in the interpretation by these two independent radiologists for each segment PDFF values. Additionally, we also have shown that the difference in the PDFF values interpreted by the two radiologists at all data points was within 95% CI (Fig 3)

**Table 2:** Interclass correlation coefficient for Histology reading by two independent readers.

Observation	Agreement between Internal Pathologists		Agreement between Internal &External Pathologists	
	Estimated coefficient of reliability, ICC (95% CI)	Estimated coefficient of within-subject variance (95% CI)	Estimated coefficient of reliability, ICC (95% CI)	Estimated coefficient of within-subject variance (95% CI)
**Macro-steatosis**	0.89 (0.77, 0.95)	0.56 (0.27, 1.20)	0.46 (0.25, 0.69)	1.33 (0.73, 2.42)
**Micro-steatosis**	0.98 (0.95, 0.99)	0.21 (0.11, 0.39)	0.84 (0.71, 0.91)	0.68 (0.36, 1.28)
**Macro+Micro-steatosis**	0.99 (0.97, 0.99)	0.14 (0.08, 0.26)	0.99 (0.97, 0.99)	0.14 (0.08, 0.26)

**Table 3:** Interclass correlation coefficient for MRI reading by two independent readers.

Anatomic Segments	Agreement between Radiologist	
	Estimated coefficient of reliability, ICC (95% CI)	Estimated coefficient of within-subject variance (95% CI)
Segment I	0.71 (0.51, 0.86)	0.26 (0.19, 0.35)
Segment II	0.69 (0.47, 0.85)	0.25 (0.19, 0.34)
Segment III	0.75 (0.55, 0.88)	0.22 (0.16, 0.30)
Segment IVA	0.82 (0.66, 0.91)	0.20 (0.15, 0.27)
Segment IVB	0.89 (0.79, 0.95)	0.18 (0.13, 0.24)
Segment V	0.85 (0.71, 0.93)	0.20 (0.14, 0.27
Segment VI	0.55 (0.30, 0.77)	0.29 (0.22, 0.40)
Segment VII	0.77 (0.59, 0.89)	0.20 (0.15, 0.27)
Segment VIII	0.85 (0.72, 0.93)	0.18 (0.13, 0.25)

### Post-transplant outcome based on fat quantification by histology and MR PDFF

Pearson product-moment correlation coefficient was computed to assess the relationship between MRI-PDFF %, histology (macro-steatosis %), and liver function tests at 24 hours, 48 hours, 72 hours, 1 week, 2 weeks, and 4 weeks. Both histology and MRI-PDFF showed a positive correlation (Pearson's correlation coefficient) with the bilirubin level in the immediate post-transplant period (Table 4 and Table 5).The correlation was particularly strong at 1 week after LT with a stronger correlation for histology read out by the pathologists (External Pathologist: r = 0.75, p<0.0001; Internal Pathologist A: r = 0.59, P = 0.003; Internal Pathologist B: r = 0.72, P = 0.0001, Table 4) compared to correlation with MRI PDFF (Radiologist A: r = 0.51, P = 0.005; Radiologist B: r = 0.52, P = 0.005, Table 5). A moderately strong correlation was also noted for INR at 1 week after LT for histology as well as MRI PDFF (Tables 4 and 5). However, no significant/consistent correlation was noted with other measures of liver function tests. Additionally, there was a moderately strong correlation was noted for the length of ICU stay at 1 week after LT for histology although the association was less strong with MRI PDFF (Table 4 and 5). No correlation was noted for the length of hospitalization with either MRI PDFF or %steatosis on histology.

**Table 4:** Correlations of early post-transplant liver chemistry, and clinical outcomes based on hepatic fat quantification by histology.

Parameters	HISTOLOGY					
	External Pathologist		Internal Pathologist A		Internal Pathologist B	
	Pearson correlation coefficient	P	Pearson correlation coefficient	P	Pearson correlation coefficient	P
Bilirubin at 24 hrs (mg/dL)	0.39	0.03	0.34	0.10	0.43	0.04
Bilirubin at 48 hrs (mg/dL)	0.56	0.002	0.41	0.048	0.54	0.008
Bilirubin at 72 hrs (mg/dL)	0.50	0.006	0.38	0.07	0.53	0.001
Bilirubin at 1 week (mg/dL)	0.75	< .0001	0.59	0.003	0.72	0.0001
Bilirubin at 2 weeks (mg/dL)	0.44	0.02	0.27	0.21	0.22	0.32
Bilirubin at 4 weeks (mg/dL)	0.22	0.27	0.22	0.31	0.34	0.11
ALT at 24 hours (IU/mL)	0.10	0.61	0.25	0.25	0.22	0.32
ALT at 48 hours (IU/mL)	0.19	0.33	0.32	0.13	0.34	0.11
ALT at 72 hours (IU/mL)	0.12	0.53	0.24	0.26	0.27	0.22
ALT at 1 week (IU/mL)	0.01	0.93	0.14	0.50	0.14	0.54
ALT at 2 weeks (IU/mL)	-0.10	0.61	0.13	0.55	0.11	0.63
ALT at 4 weeks (IU/mL)	0.17	0.39	-0.01	0.96	0.04	0.87
AST at 24 hours (IU/mL)	0.37	0.05	0.59	0.003	0.56	0.005
AST at 48 hours (IU/mL)	0.38	0.04	0.41	0.054	0.50	0.01
AST at 72 hours (IU/mL)	0.22	0.29	0.29	0.17	0.35	0.10
AST at 1 week (IU/mL)	0.29	0.14	0.33	0.13	0.34	0.11
AST at 2 weeks (IU/mL)	-0.11	0.61	0.02	0.93	0.06	0.81
AST at 4 weeks (IU/mL)	-0.12	0.56	0.03	0.88	0.10	0.64
ALP at 24 hours (IU/mL)	-0.15	0.43	0.05	0.83	-0.06	0.79
ALP at 48 hours (IU/mL)	0.06	0.75	0.54	0.007	0.48	0.02
ALP at 72 hours (IU/mL)	0.32	0.09	0.48	0.02	0.43	0.03
ALP at 1 week (IU/mL)	0.15	0.46	0.33	0.12	0.27	0.21
ALP at 2 weeks (IU/mL)	-0.16	0.41	-0.002	0.99	-0.08	0.73
ALP at 4 weeks (IU/mL)	-0.21	0.29	0.03	0.89	0.12	0.58
INR at 24 hours (IU/mL)	0.42	0.03	0.37	0.09	0.48	0.02
INR at 48 hours (IU/mL)	0.33	0.09	0.33	0.12	0.44	0.04
INR at 72 hours (IU/mL)	0.23	0.24	0.21	0.32	0.30	0.16
INR at 1 week (IU/mL)	0.43	0.024	0.42	0.045	0.51	0.01
INR at 2 weeks (IU/mL)	0.01	0.93	0.0008	0.99	0.03	0.89
INR at 4 weeks (IU/mL)	-0.05	0.75	-0.006	0.97	0.07	0.74
Length of ICU stay (Days)	0.50	0.007	0.42	0.048	0.56	0.006
Length of Hospitalization	0.22	0.25	0.26	0.24	0.40	0.057

**Table 5:** Correlations of early post-transplant liver chemistry, and clinical outcomes based on hepatic fat quantification by MRI PDFF.

Parameters	MRI PDFF			
	Radiologist A		Radilologist B	
	Pearson correlation coefficient	P	Pearson correlation coefficient	P
Bilirubin at 24 hrs (mg/dL)	0.33	0.08	0.28	0.15
Bilirubin at 48 hrs (mg/dL)	0.33	0.08	0.31	0.10
Bilirubin at 72 hrs (mg/dL)	0.34	0.07	0.34	0.07
Bilirubin at 1 week (mg/dL)	0.51	0.005	0.52	0.005
Bilirubin at 2 weeks (mg/dL)	0.23	0.24	0.26	0.18
Bilirubin at 4 weeks (mg/dL)	0.11	0.56	0.12	0.53
ALT at 24 hours (IU/mL)	0.09	0.63	0.08	0.68
ALT at 48 hours (IU/mL)	0.14	0.47	0.17	0.40
ALT at 72 hours (IU/mL)	0.08	0.67	0.11	0.59
ALT at 1 week (IU/mL)	0.004	0.98	-0.0006	0.99
ALT at 2 weeks (IU/mL)	-0.07	0.73	-0.09	0.64
ALT at 4 weeks (IU/mL)	-0.18	0.37	-0.18	0.36
AST at 24 hours (IU/mL)	0.20	0.29	0.26	0.18
AST at 48 hours (IU/mL)	0.20	0.31	0.28	0.14
AST at 72 hours (IU/mL)	0.09	0.66	0.16	0.43
AST at 1 week (IU/mL)	0.27	0.17	0.26	0.19
AST at 2 weeks (IU/mL)	-0.15	0.47	-0.18	0.38
AST at 4 weeks (IU/mL)	-0.19	0.34	-0.19	0.34
ALP at 24 hours (IU/mL)	0.09	0.64	0.13	0.52
ALP at 48 hours (IU/mL)	0.11	0.57	0.05	0.80
ALP at 72 hours (IU/mL)	0.43	0.02	0.43	0.03
ALP at 1 week (IU/mL)	0.24	0.20	0.17	0.39
ALP at 2 weeks (IU/mL)	-0.17	0.40	-021	0.30
ALP at 4 weeks (IU/mL)	-0.04	0.83	-0.10	0.60
INR at 24 hours (IU/mL)	0.42	0.03	0.40	0.04
INR at 48 hours (IU/mL)	0.35	0.07	0.32	0.13
INR at 72 hours (IU/mL)	0.13	0.51	0.18	0.38
INR at 1 week (IU/mL)	0.50	0.008	0.50	0.008
INR at 2 weeks (IU/mL)	0.002	0.99	-0.01	0.95
INR at 4 weeks (IU/mL)	0.01	0.96	-0.03	0.89
Length of ICU stay (Days)	0.37	0.051	0.46	0.01
Length of Hospitalization	0.02	0.91	0.09	0.64

We performed a sensitivity analysis by categorizing the patients based on histology (<20% versus ≥20%), and we noted there was a trend for increased total bilirubin level at 1-week post-transplant in the high steatosis group (10.9± 9.6 vs. 2.7± 2.3., p = 0.051). Mean total bilirubin peaked at a much higher level in the high steatosis group (≥20% on histology) and remained persistently higher even up to 2 months post LT (S7 Fig). In general, there were no significant differences in the AST, ALT, ALP, and INR at 1 week after liver transplant. However, we noted a higher peak level of AST and ALT in the group with ≥20% allograft steatosis on histology in the immediate post LT period (S7 Fig). Length of ICU stay, length of hospitalizations, re-explorations, re-admissions were not different.

When we analyzed our data based on fat fraction measurement using MRI PDFF (< 10% versus ≥ 10%), we noted a similar significantly higher total bilirubin level at 1-week post LT in subjects with PDFF % ≥10% (13±10.5 vs.2.95±2.6 vs., p = 0.026). Mean bilirubin remained persistently higher in the group with high PDFF up to 2 months post LT (S7 Fig). Again, we noted a higher peak level of AST, and ALT in the group with ≥10% allograft steatosis on MRI PDFF in the immediate post LT period (S7 Fig). Length of ICU stay, length of hospitalizations, re-explorations, re-admissions were not different.

### Post-transplant early allograft dysfunction based on fat quantification by histology and MR PDFF

**Fig 4:**
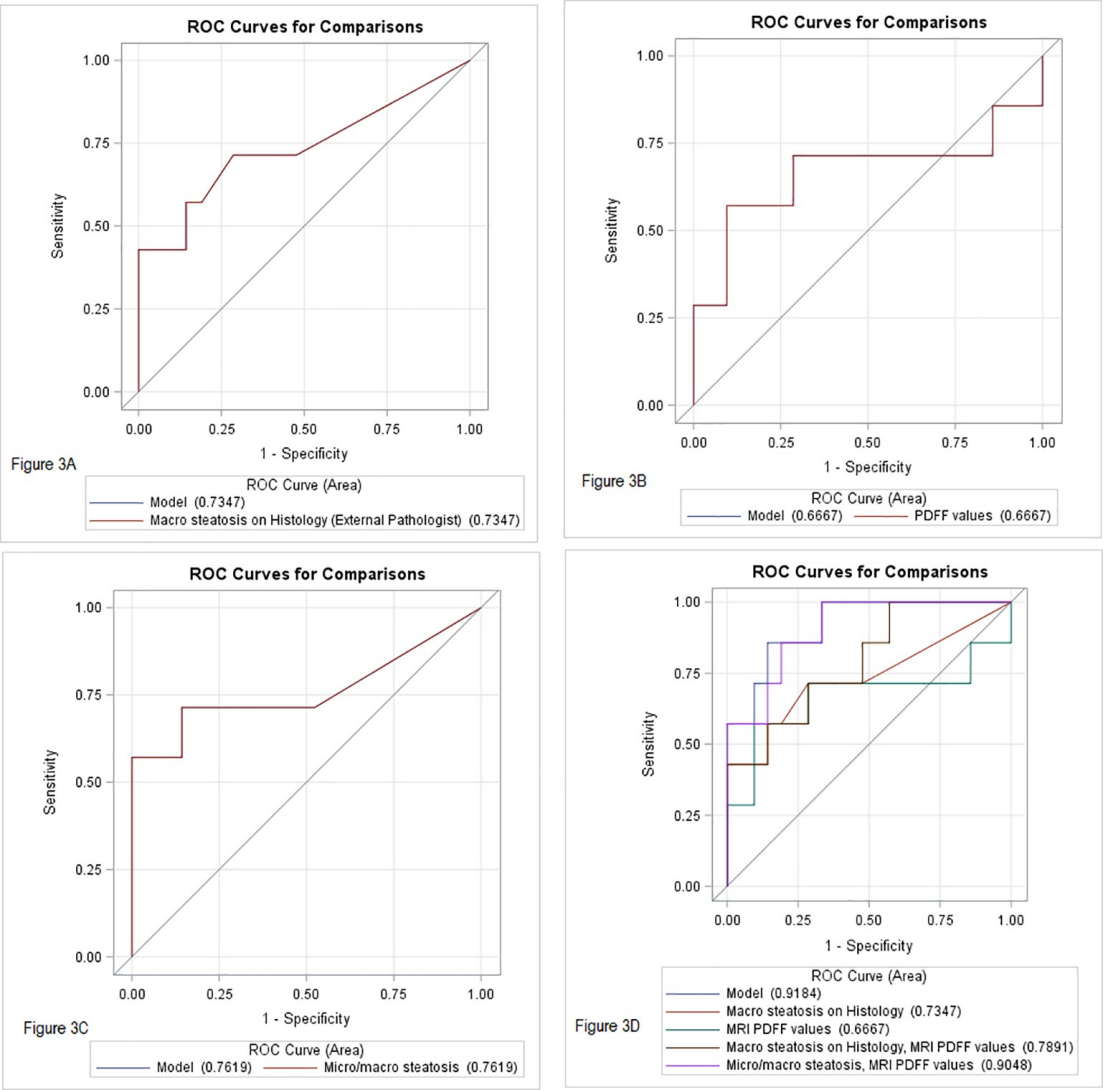
Comparison of area under ROC curves in predicting early allograft dysfunction based on degree of hepatic steatosis (external pathologist- read out). Results are compared for hepatic steatosis quantification based on histology and MRI PDFF measurements.

EAD was noted in 7(25%) subjects in the current cohort of patients. EAD findings of all the subjects enrolled in the final cohort is summarized in S1 Table. A comparison of area under ROC curves in predicting early allograft dysfunction based on the degree of hepatic steatosis as read by the external pathologist is presented in Fig 4. AUC for histology determined macro steatosis (per external pathologist) to predict EAD was 73% (95% CI: 48–99). Similarly, AUC for macro steatosis read out by Internal pathologist A for predicting EAD was 80% (95% CI: 57–100), and for Internal pathologist B for predicting EAD was 80% (95% CI: 57–100). AUC for histology determined micro plus macro steatosis by the external pathologist to predict EAD was 76% (95% CI: 49–100). ROC association using MRI PDFF to predict EAD showed an AUC of 67(35–98) only. Comparison of the ROC curves in a multivariate model revealed, adding MRI PDFF values to macro steatosis increased ability of the model in predicting EAD (AUC: 79%, 95% CI: 59–99), and addition of macro plus micro steatosis based on histology predicted EAD significantly better (AUC: 90%: 79–100, P = 0.054).

**Fig 5:**
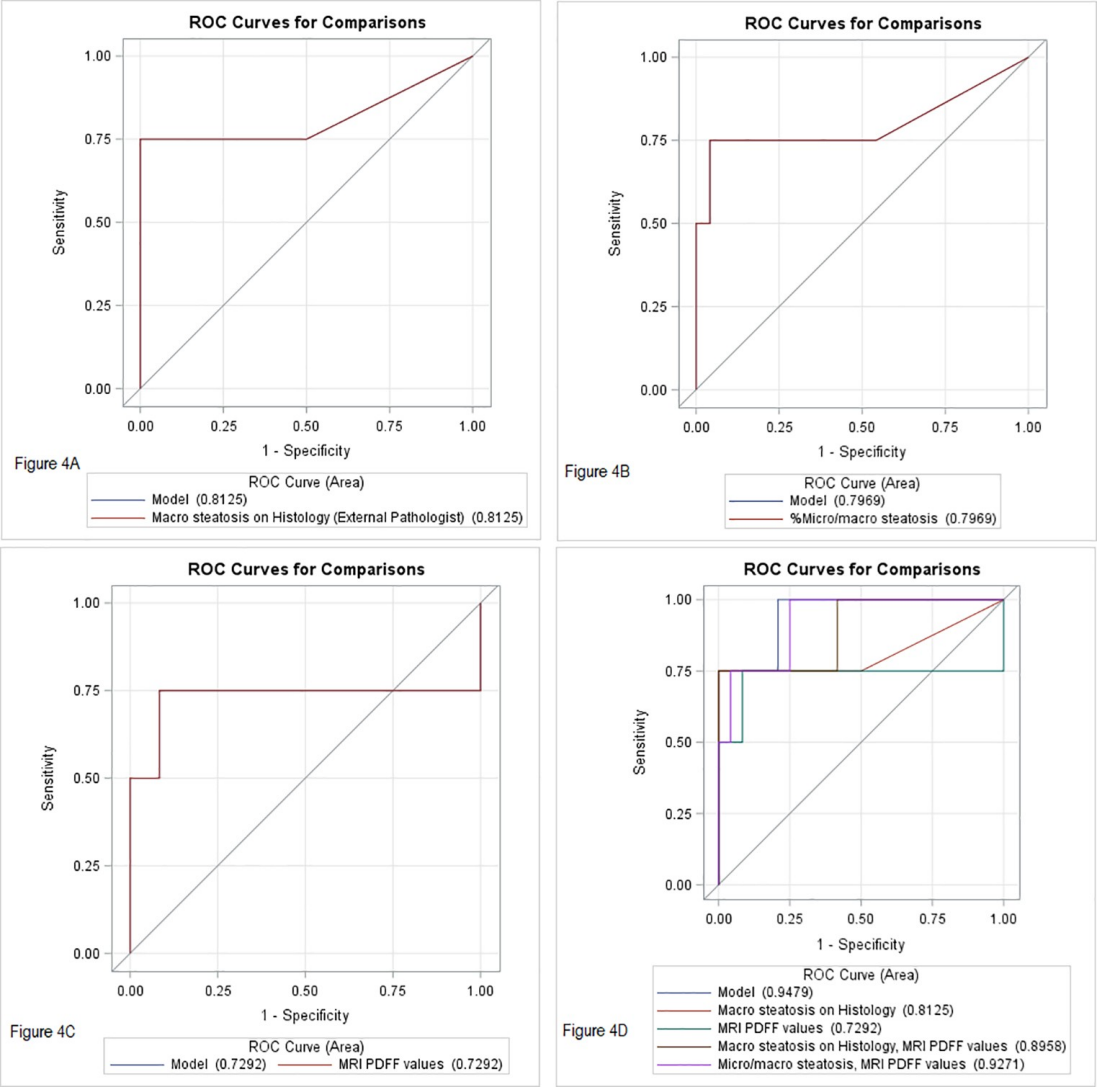
Comparison of area under ROC curves in predicting peak bilirubin > 10 mg/dl. Results are compared for hepatic steatosis quantification based on histology (External Pathologist -read out) and MRI PDFF measurements.

We also performed a subset analysis to predict peak bilirubin > 10 mg/dl at 1 week (Fig 5). Again, we noted AUC for histology determined macro steatosis read out by external pathologist to predict peak bilirubin > 10 mg/dl was 73% (95% CI: 25–100). Similarly, these results for Internal pathologist A was 80% (95% CI: 48–100), and for internal pathologist B was 82% (95% CI: 51–100), AUC for histology determined micro plus macro steatosis read out by external pathologist to predict peak bilirubin > 10 mg/dl was 80% (95% CI: 42–100). Similar results were obtained for internal pathologist A (79%; 95% CI: 45.50–100), and internal pathologist B ((79%; 95% CI: 45–100). ROC association using MRI PDFF to predict peak bilirubin > 10 mg/dl showed an AUC of 73(25–100) only. Comparison of the ROC curves in a multivariate model revealed, adding MRI PDFF values increased overall performance of the model in predicting peak bilirubin > 10 mg/dl for both macro-steatosis (AUC: 90, 95% CI: 69–100) as well as of macro plus micro steatosis (AUC: 93%: 80–100) compared to MRI PDFF values alone.

## Discussion

In this pilot study, we have demonstrated a similar degree accuracy of *ex-vivo* MRI-PDFF imaging of donor liver enroute to the operating room in quantifying hepatic steatosis, and as an adjunct tool for assessment of hepatic steatosis to liver biopsy, thus potentially providing an additional safety net in selecting quality donor liver with optimal hepatic steatosis. Additionally, we have demonstrated the potential utility of MRI‐PDFF as an imaging biomarker for predicting early allograft dysfunction related to severe steatosis. To our knowledge, this is the first study demonstrating the potential utility of ex-vivo MRI imaging for donor liver selection.

Assessment of liver allograft steatosis is an important first step in the selection of the suitability of donor organs. Historically, steatosis has been classified as microvesicular or macrovesicular, based on hepatocyte fat droplet size and nucleus displacement. Microvesicular steatosis manifests histologically as diffuse deposition of small lipid droplets in the hepatocyte cytoplasm with a resulting foamy appearance. On the other hand, in the setting of macrovesicular steatosis, two types of fat droplets are seen, small droplets and large droplet. Fat droplets in small droplet steatosis are not large enough to displace the nucleus, and often this finding is misreported in the literature as being microvesicular steatosis on biopsy leading to confusion[9]. We suspect these differences in the reporting may have affected the moderate disagreement in the assessment of the degree of macro-steatosis among the internal and external (procurement site) pathologist. This is evident with little, if any, disagreement among the readers when we combined the macro and micro-steatosis (Table 2).

Using allografts pre-selected for LT, we have shown strong correlation (defined as *r* = 0.70 to 0.89) of MRI-PDFF with allograft steatosis on histology as assessed on procurement site as well as on secondary readouts by two independent expert liver pathologist suggesting its potential utility in allograft section. Several in vivo studies have compared MRI-PDFF or histologic evaluation from a single biopsy showing a good correlation[10–13]. In a study using explanted human liver, Bannas et al have shown MRI-PDFF as an accurate, precise, and reader-independent noninvasive imaging biomarker of liver triglyceride content, capable of steatosis quantification over the entire liver[14]. Bannas et al specifically demonstrated that MRI-PDFF had higher intraobserver and interobserver agreement and higher precision (repeatability) than histologic steatosis grading. Additionally, the utility of MRI as a modality for donor selection has been recently evaluated in 144 liver donor candidates with magnetic resonance spectroscopy (MRS) and 6-echo Dixon magnetic resonance imaging (MRI)[15]. The authors noted, MR-PDFF estimate of negligible hepatic fat percentage (<5%) has sufficient NPV for excluding clinically significant hepatic steatosis (≥10%) in living liver donor candidates obviating the need for liver biopsy.

A liver biopsy at the procurement site using frozen section specimen currently remains the gold standard in the quantification of allograft steatosis. However, frozen section assessment is not perfect, and is fraught with sampling variability, freezing artifact, and intra- and inter-observerr variability[14]. In the current study, we noted the poor quality of frozen section liver biopsy specimen in 26.1% of the cases which could very well impact accurate assessment of the steatosis. We also noted significant variability between the internal and external pathologist’s read-out on macro-steatosis, but the agreement was strong among the internal pathologists. Bannas et al have shown that that [1]repeated samples from one liver segment can vary dramatically and [2] individual liver segments might have much higher or lower steatosis degrees than the prevailing average steatosis degree[14]. Frozen section biopsy has a high positive predictive value of in detection of >50% steatosis, which could very well serve as a basis to reject the use of graft for transplantation[16]. However, a decision of graft acceptance based solely on the frozen section is difficult as the sensitivity of detecting allograft steatosis > 50% is only moderate and must also be made on consideration of other well-known factors for poor posttransplant function[16]. A precise quantification that is accurate, reliable, reproducible, and not fraught with inter/intraobserver variation is highly desirable for preventing early allograft dysfunction, and potential primary non-function of the liver. In the present study using ex-vivo MRI-PDFF imaging, we have demonstrated a strong correlation of PDFF values in human deceased liver similarly to liver biopsy suggesting its role in potential utility in future donor selection.

Several studies have shown a linear correlation of MRI-determined proton density-fat fraction with histology-determined steatosis grade in adults with NAFLD[17]. In these studies, PDFF values of about 10% or higher correlate with NASH-CRN grade 1 steatosis (5%-33%). In a study including 113 patients, 34% of subjects had steatosis grade 0 or 1 (0–33%), 39% had steatosis grade 2 (33%-66%), and 27% had steatosis grade 3 (>67%); corresponding mean PDFF values were 9.8%±3.7%, 18.1%±4.3%, and 30.1%±8.1%, respectively[18]. The % of hepatic steatosis on histology in this study is about 2–3 times the mean PDFF values. PDFF classified steatosis grade 0–1 vs 2–3 with an area under the ROC curve (AUROC) of 0.95 (95% CI, 0.91–0.98), and grade 0–2 vs grade 3 steatosis with an AUROC of 0.96 (95% CI, 0.93–0.99) [18]. Another study reported, MR imaging PDFF threshold of 6.4% to diagnose grade 1(5% to 33% steatosis) or higher steatosis, and had 86% sensitivity (71 of 83 patients; 95% confidence interval [CI]: 76, 92) and 83% specificity (five of six patients; 95% CI: 36, 100) [12]. Based on these studies, we adopted a PDFF cut off of > 10% would correspond to hepatic steatosis by histology of > 20% as clinically significant hepatic steatosis. Using these cut-off values we have noted significantly higher total bilirubin level, AST, and ALT in both subjects with PDFF % ≥10% or hepatic steatosis by histology of > 20% which even persisted up to 2 months post LT (S7 Fig). These findings need to be validated in future studies including a large number of patients.

Early allograft dysfunction identifies liver transplant allografts with initial poor function and has been shown to predict inferior post transplants allograft and patient survival[19, 20]. In the present study, we have demonstrated that MRI PDFF potentially can predict donor EAD, and future studies should be conducted to further evaluate its clinical utility. In this pilot study, MRI PDFF not only performed in predicting EAD at the same level of accuracy, but the addition of histology particularly macro/micro-steatosis improved the prediction of EAD compared to either alone. Macrovesicular steatosis has been reported to be an important risk factor for EAD after orthotopic liver transplantation (OLT), especially those with severe steatosis, and with prolonged CIT(Cold Ischemia Time)[21]. However, in selected donors with moderate macrovesicular steatotic livers can be used successfully for transplantation if the CIT is kept very short. In the present study, EAD was noted in 7(26%) subjects and correlated with the severity of donor macro-steatosis. We found, overall histology determined macrosteatosis in the donor liver performed with similar accuracy as compared to MRI determined macro-steatosis in predicting EAD. Of all the components of the EAD, total bilirubin appears to correlate significantly with the degree of hepatic steatosis. Both MRI PDFF values and hepatic steatosis based on histology strongly predicted peak bilirubin > 10 mg/dl at week after LT an important marker for early allograft dysfunction. Besides, the peak levels of AST, ALT, and ALP were much higher in the donor with significant steatosis (≥ 20% on histology, or ≥10% on MRI PDFF).

Despite encouraging results, the current study should be considered as a proof of concept rather than a recommendation given this is a pilot study, and the results are based on a small number of patients. This study could serve as a basis for future studies looking into the utility of ex vivo MRI PDFF in screening high-risk donor liver with donor steatosis > 20% on histology in reassessing hepatic fat content. If the results are validated, this approach could potentially serve to expand the donor pool by either increased acceptance by the procuring surgeon in the event of overestimation of the donor steatosis by histology or might results in better donor selection by potentially averting disastrous consequences of primary non-function in the event of underestimation of hepatic steatosis. Secondly, the number of DCD (Donation after Cardiac Death) in the current cohort was significantly high (> 37%) compared to the national average, which reflects the unique practice of our center, which may have affected the overall results of higher early allograft dysfunction. As such, the results may not be generalizable to other centers; further, validation is needed. Third, MRI PDFF can potentially overestimate hepatic steatosis as it measures both macro and micro steatosis, and as such the predictive ability for EAD based on this technique needs further validation as prior studies have shown grade of hepatic micro-steatosis does not impact postoperative outcome after liver transplantation [22]. Additionally, the temperature of the UW solution (University of Wisconsin solution) may have affected the MRI-PDFF outcome, an issue not addressed in the current study. Also, the additional cost and logistics associated with this approach as compared to histology need to be addressed in future studies. Finally, the inter-observer variability in the current study related to the variability of interpretation by a different pathologist at a different procuring site may have affected the accuracy of the histological interpretation. However, the data on liver biopsy is collected in real-time in the current study which has practical implication as reading by a single pathologist may not be practically possible for donor selection due to time constraints and potentially increasing cold ischemia time. The median MRI scan time and the median time from MRI to organ-in-room time was 2 and 11 minutes, suggesting if properly coordinated this approach is feasible without significantly increasing CIT. As such, the addition of MR PDFF may have practical utility as a second-line tool for additional screening for steatosis considering potential sampling variability in histopathology which is less likely with MRI.

In conclusion, in this proof of concept study, we have demonstrated that ex-vivo MRI-PDFF imaging quantifies hepatic steatosis in human deceased liver similarly to liver biopsy, and is less likely to be associated with significant variability in reading and could therefore potentially serve as a noninvasive marker of early allograft dysfunction related to severe graft steatosis. Future prospective studies should be performed in marginal steatotic donor livers to better define utility of MR PDFF values in donor selection, and in predicting EAD.

## Supporting information

S1 Fig Fig 2A. Correlation of MRI-PDFF with histology (Internal Pathologist A- read out) when only macro steatosis was included.
(TIF)

S2 Fig Correlation of MRI-PDFF with histology (Internal Pathologist B- read out) when only macro steatosis was included. (TIF)

S3 Fig Correlation of MRI-PDFF with histology (Internal Pathologist A-read out) when macro plus micro steatosis was included. (TIF)

S4 Fig Correlation of MRI-PDFF with histology (Internal Pathologist B-read out) when macro plus micro steatosis was included. (TIF)

S5 Fig Fit plot for MRI-PDFF compared with histology (External Pathologist- read out) when only macro steatosis was included. (TIF)

S6 Fig Fit plot for MRI-PDFF compared with histology (External Pathologist- read out) when macro plus micro steatosis was included. (TIF)

S7 Fig Post-transplant Bilirubin, AST, and ALP levels compared based on degree of hepatitis steatosis as measured by histology read-out by external pathologist (grouped by < 20% hepatic steatosis vs >/ = 20% hepatic steatosis) and by PDFF values (<10% PDFF% vs >/ = PDFF values). (TIF)

S1 Table (XLS)

S1 Data (DOCX)
