# Supplementary figures and images for: A pilot study of ex-vivo MRI-PDFF of donor livers for assessment of steatosis and predicting early graft dysfunction
Source: PLoS One. 2020 May 14;15(5):e0232006. doi: 10.1371/journal.pone.0232006 (PMC7224456; doi:10.1371/journal.pone.0232006)

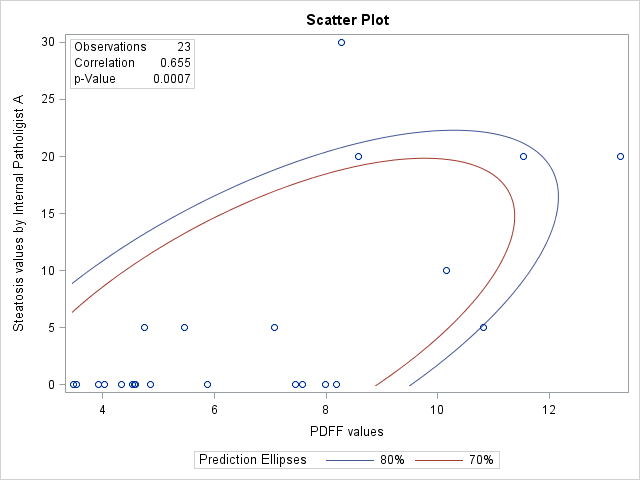

Supplement: S1 Fig — Correlation of MRI-PDFF with histology (Internal Pathologist A- read out) when only macro steatosis was included. (TIF) [file pone.0232006.s001.tif]

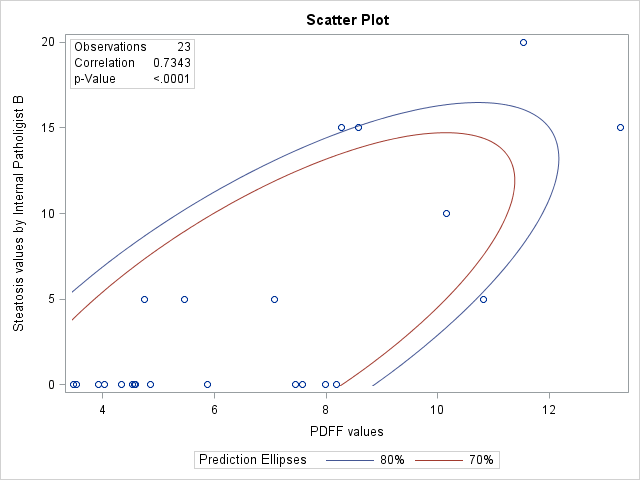

Supplement: S2 Fig — (TIF) [file pone.0232006.s002.tif]

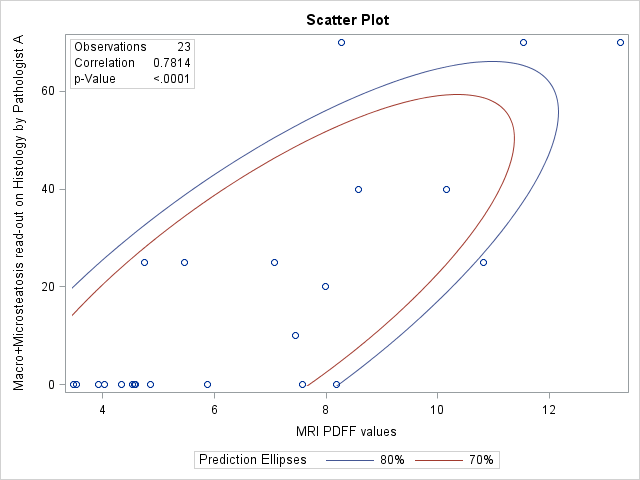

Supplement: S3 Fig — (TIF) [file pone.0232006.s003.tif]

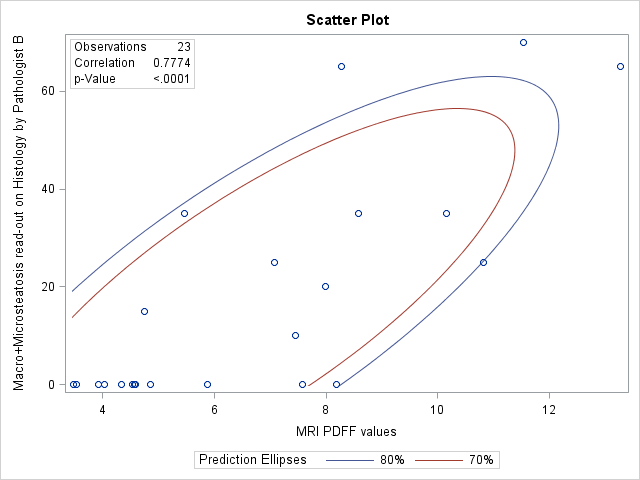

Supplement: S4 Fig — (TIF) [file pone.0232006.s004.tif]

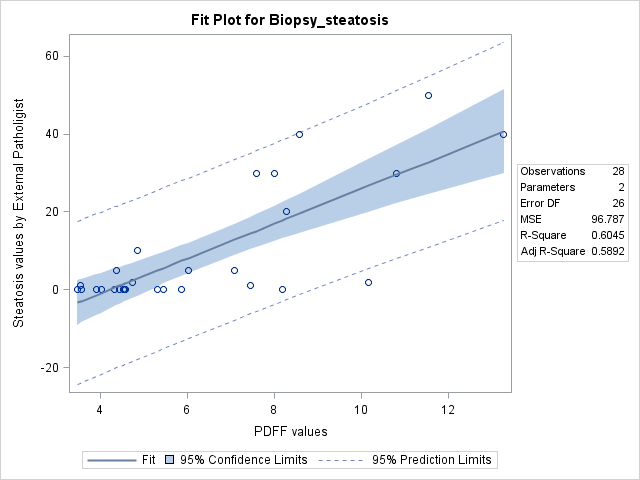

Supplement: S5 Fig — (TIF) [file pone.0232006.s005.tif]

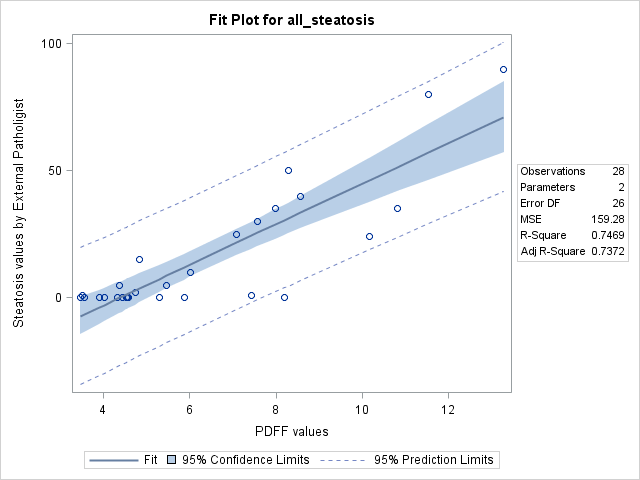

Supplement: S6 Fig — (TIF) [file pone.0232006.s006.tif]

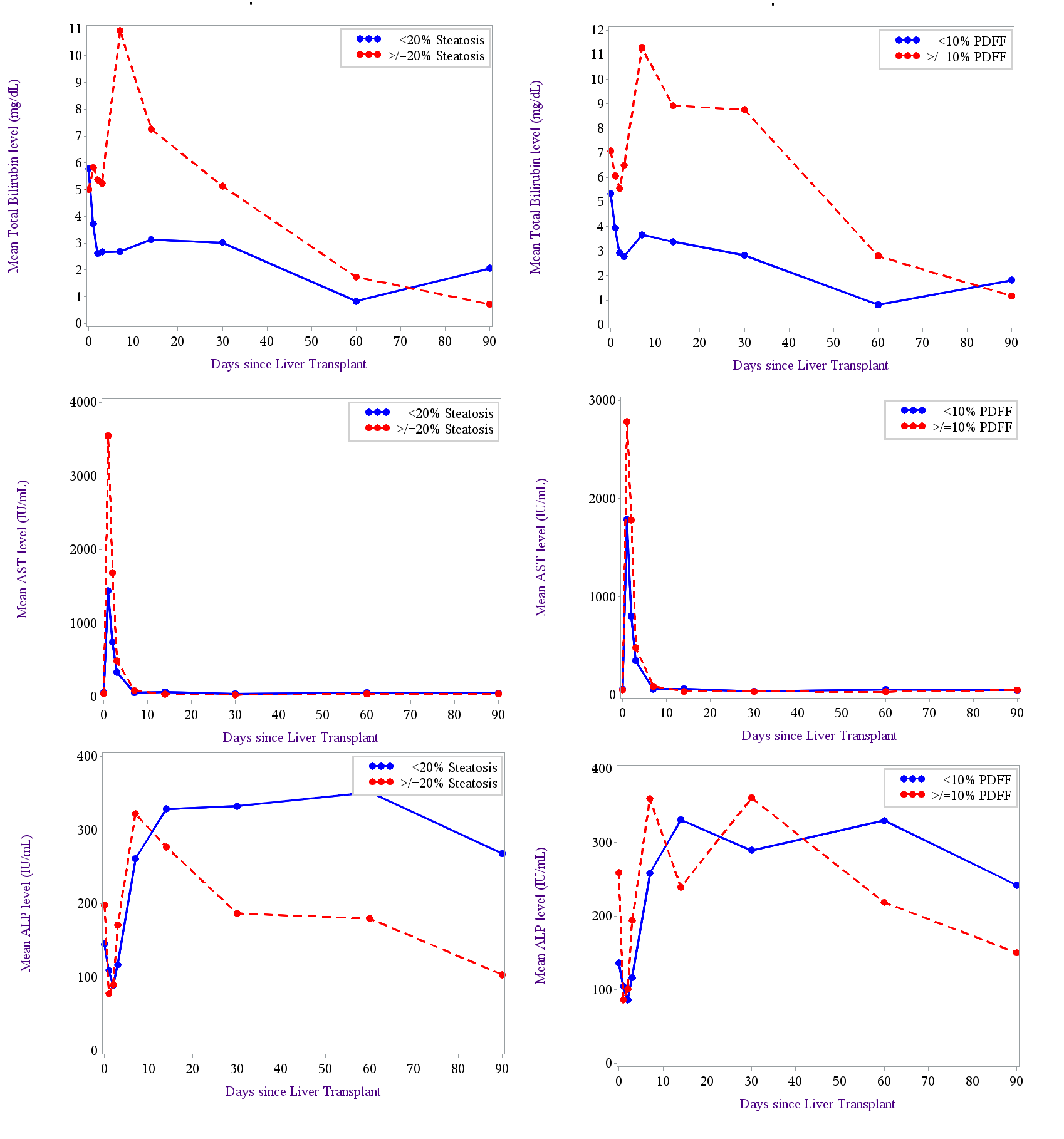

Supplement: S7 Fig — (TIF) [file pone.0232006.s007.tif]
