# Supplementary material for: A pilot study of ex-vivo MRI-PDFF of donor livers for assessment of steatosis and predicting early graft dysfunction
Source: PLoS One. 2020 May 14;15(5):e0232006. doi: 10.1371/journal.pone.0232006 (PMC7224456; doi:10.1371/journal.pone.0232006)
Supplement: S1 Data — (DOCX) [file pone.0232006.s009.docx]

Additional MRI scanner parameters :

Imaging was performed at 1.5T on GE Optima MR450w.

Axial T2 single shot fast spin echo sequences (SSFSE) were utilized for anatomic correlation with PDFF maps

Slice thickness 8 mm

TR 650

TE 91

Echo Train Length 1

Flip angle 90

HD 32 channel body coil

PDFF sequences

Slice thickness 10 mm

TR 300

TE 1.7 – 28.4

Echo Train Length 6

Flip angle 8

HD 32 channel body coil
